# Use, Concerns, and Perspectives on AI in Health Care Among French Health Professionals and Students: Web-Based Cross-Sectional Survey

**DOI:** 10.2196/89152

**Published:** 2026-08-05

**Authors:** Aurelia Alati, Grégoire Pigné, Carole-Anne Brugère, Jean-Emmanuel Bibault

**Affiliations:** 1 Department of Radiation Oncology, Université Paris Cité, Hôpital Européen Georges-Pompidou, 20 rue Leblanc, Paris, Île-de-France, 75015, France, 33 783499288; 2 Inserm UMRS 1138, Centre de Recherche des Cordeliers, Paris, Île-de-France, France; 3 Department of Radiation Oncology, Institut de Cancérologie et d'Hématologie Universitaire de Saint-Étienne, Saint-Priest-en-Jarez, Saint-Etienne, France; 4 PulseLife, Lyon, France

**Keywords:** artificial intelligence, AI, health care professionals, students, medical education, digital health, survey, training needs

## Abstract

**Background:**

AI is increasingly discussed and deployed in health care, yet safe and effective implementation depends on the preparedness, trust, and training of the professionals who are expected to use these tools.

**Objective:**

This study aimed to assess current AI use, perceived benefits and concerns, confidence, and training needs among French health care professionals and students.

**Methods:**

We conducted a national web-based cross-sectional survey distributed through the PulseLife professional community between December 4, 2024, and March 5, 2025. The survey instrument was administered in French and included respondent characteristic items together with 12 substantive closed-ended questions covering current AI use, confidence, perceived benefits, and concerns, and interest in AI-related training. Access was restricted to authenticated individual PulseLife accounts, and multiple submissions from the same account were not allowed. Questions were not mandatory; incomplete questionnaires were retained for item-level analyses, and percentages were calculated using item-specific denominators. Because the exact invitation denominator was not retained by the platform, view, participation, and completion rates could not be calculated. Descriptive statistics and Pearson chi-square tests were performed using R. Internal consistency and exploratory psychometric properties were assessed using the Cronbach α, exploratory factor analysis, and confirmatory factor analysis.

**Results:**

A total of 1625 respondents participated, including 1212 (74.6%) health professionals and 413 (25.4%) students. Among professionals, physicians represented the largest group (642/1212, 53%), followed by nurses (232/1212, 19.1%) and pharmacists (92/1212, 7.6%). Only 6.6% (90/1366) of the respondents reported prior AI-specific training, whereas 78.3% (920/1175) wished to receive such training. Confidence in AI for diagnosis and patient management remained limited: only 9.2% (120/1301) of the respondents reported being very confident. Nearly half (673/1455, 46.3%) of the respondents who answered this item reported no current AI use in professional activity, whereas 10.5% (153/1455) reported frequent use. Physicians and younger respondents reported more frequent AI use, and prior AI training was associated with greater confidence (*P*<.001 in all cases). Commonly perceived benefits included improved diagnosis (774/1625, 47.6%), time savings (685/1625, 42.2%), reduced medical errors (634/1625, 39%), and improved patient follow-up (593/1625, 36.5%). Frequently reported concerns included algorithmic bias (785/1625, 48.3%), limited transparency (666/1625, 41%), deterioration of the patient–health care professional relationship (628/1625, 38.6%), and data confidentiality (557/1625, 34.3%).

**Conclusions:**

In this national French sample, formal AI training was uncommon despite high interest in receiving it. These findings support the need for more structured educational initiatives in AI literacy across undergraduate, postgraduate, and continuing professional education. Because this study relied on a convenience sample recruited through a digital platform, the findings should be interpreted as descriptive and exploratory rather than nationally representative.

## Introduction

AI is increasingly being integrated into health care, with applications ranging from clinical decision support and imaging analysis to workflow optimization, administrative automation, and patient monitoring. Safe implementation depends not only on technical performance but also on the preparedness, trust, and training of the professionals expected to use these tools [1,2]. In France, AI has been identified as a strategic priority for health innovation, yet the practical readiness of frontline professionals and students remains insufficiently documented [1].

International studies suggest that interest in AI frequently exceeds formal preparation. In a web-based survey of 293 physicians and medical students, AlZaabi et al [3] found generally positive attitudes toward AI but also a clear need for education, with 69% of participants reporting that AI was an emerging field they wished to learn more about. In Spain, Catalina et al [4] reported that implementation in primary care depended not only on technical feasibility but also on professionals’ knowledge and acceptance of AI. In Germany, Laupichler et al [5] showed among 377 medical students that prior AI education and interest in AI were both associated with higher AI literacy. More recently, a Saudi Arabian survey of 1221 medical and health sciences students found moderate AI readiness overall, and 44.5% of respondents believed that AI-related courses should be mandatory [6].

The educational implications of these findings are increasingly emphasized in the literature. A 2024 scoping review identified 21 eligible papers describing 30 educational programs and only 2 curriculum frameworks for AI across medical students, residents, and practicing physicians, underscoring how fragmented the field remains [7]. Stakeholder and curriculum studies have further emphasized that AI competencies should extend beyond technical understanding to include ethical reasoning, implementation awareness, and critical appraisal [8-10]. Against this background, the objective of this study was to assess current use of AI, perceived benefits and concerns, confidence, and training needs among French health care professionals and students, with direct relevance for curriculum design, continuing professional development, and the responsible integration of AI into clinical practice.

## Methods

### Study Design and Setting

We conducted a national web-based cross-sectional survey of health care professionals and students in France. The survey was disseminated through PulseLife, a digital platform and professional community used by health care workers in France. Recruitment was conducted via email between December 4, 2024, and March 5, 2025.

No financial incentive was offered. The exact number of invited individuals, page view rates, participation rates, and completion rates was not retained by the platform in a form that allowed for retrospective reconstruction; accordingly, a formal response rate could not be calculated. This study should therefore be interpreted as a convenience-sample survey rather than a probability-based national survey.

### Participants, Eligibility, and Survey Administration

Eligible participants were health care professionals directly involved in care delivery and health profession students training in France. The survey was accessed through authenticated individual PulseLife accounts. Each account could submit the questionnaire only once, and multiple submissions from the same account were not allowed.

The survey instrument included demographic and professional characteristic items together with 12 substantive closed-ended questions and was administered in French. Questions were not technically mandatory, and respondents could skip items. The archived platform configuration did not retain sufficiently detailed information to reconstruct whether back navigation to earlier items was enabled. Incomplete questionnaires were retained in the analytic dataset; all percentages were therefore calculated using the number of respondents to each item as the denominator unless otherwise specified. For multi-selection items, totals could exceed 100%.

### Study Objectives

The primary objective was to assess use, concerns, and perspectives regarding AI technologies among French health care professionals and students. Secondary objectives were to explore associations between respondent characteristics and AI use or confidence and identify training needs with direct relevance to medical and health professions education.

### Questionnaire Development and Psychometric Assessment

The questionnaire covered respondent characteristics and AI use, confidence, perceived benefits, perceived concerns, and training interest (Multimedia Appendix 1). In this survey, “AI-based solutions” was used as an umbrella term encompassing, for example, decision support systems, imaging analysis tools, administrative automation, and generative AI–enabled information tools. The questionnaire was developed through iterative multidisciplinary review by clinicians involved in AI-related clinical practice and digital health implementation. The development process aimed to ensure face validity and practical relevance for health care professionals and students in France. Before dissemination, the questionnaire was pilot-tested with 20 health care professionals and students to assess clarity, wording, and feasibility. This pilot stage was intended as a face validity and usability check rather than formal stand-alone validation.

Because the instrument was created for this study, we report its psychometric properties as exploratory support rather than definitive external validation. Internal consistency across domains was acceptable (Cronbach α=0.76-0.84). Sampling adequacy was supported by a Kaiser-Meyer-Olkin index of 0.83, and the Bartlett test of sphericity was significant (*P*<.001). Exploratory factor analysis supported a 3-factor structure (concerns, perceived benefits, and assisted tasks), a Confirmatory factor analysis of the 14-item, 3-factor model indicated acceptable fit (χ²/df=2.1; df=74; comparative fit index=0.94; Tucker-Lewis index=0.92; root mean square error of approximation=0.05; standardized root mean square residual=0.06). Known group validity was supported by higher AI confidence among physicians, younger respondents, and participants with prior AI training. More detailed factor analytic parameters, including extraction method, rotation strategy, factor retention criteria, item retention thresholds, and confirmatory factor analysis estimator specifications, were not retained in a form that allowed for full retrospective reporting. Similarly, domain scores were interpreted at the construct level, but a formal scoring algorithm for external reuse was not predefined because the instrument was designed for this exploratory survey. Because exploratory and confirmatory analyses were conducted on the same overall dataset, these findings should be interpreted cautiously and are further discussed as a limitation. Additional psychometric details are provided in Multimedia Appendix 1.

### Statistical Analysis

Descriptive statistics are reported as counts and percentages. Associations between categorical variables were explored using Pearson chi-square tests. No formal correction for multiple testing was applied because the inferential analyses were exploratory. This approach increases the risk of type I error, and reported associations should therefore be interpreted as hypothesis generating. Effect sizes such as the Cramér *V* were not available from the archived analytical outputs in a form that allowed for reliable retrospective reporting across all comparisons. For this reason, we retained a conservative presentation of the inferential analyses and limited their interpretation accordingly. Analyses were conducted using R (version 4.3.1; R Foundation for Statistical Computing). No weighting was applied because sampling probabilities and suitable calibration margins were unavailable.

### Ethical Considerations

This study was conducted in accordance with the Declaration of Helsinki and the French legal framework applicable to health research and personal data protection. The protocol was reviewed and approved by the local ethics committee of Hôpital Européen Georges Pompidou, Paris, France; however, no formal approval number was assigned. Participation was voluntary, and completion of the online questionnaire was considered to constitute informed consent. The dataset used was deidentified before analysis. No directly identifying information was collected in the study dataset used by the academic investigators. Participants received no financial compensation.

## Results

A total of 1625 respondents were included in the survey, including 1212 (74.6%) health professionals and 413 (25.4%) students. Among professionals, physicians represented the largest group (642/1212, 53%), followed by nurses (232/1212, 19.1%) and pharmacists (92/1212, 7.6%). Among students, medical students represented 59.1% (244/413) of the respondents, followed by nursing students (69/413, 16.7%). Most professionals reported working in hospitals (429/1212, 35.4%), private practice (308/1212, 25.4%), or private clinics (123/1212, 10.1%; Table 1).

**Table 1.** Population characteristics.

Characteristic	Participants, n (%)
Health professionals (n=1212)	
Physicians	642 (53)
Nurses	232 (19.1)
Pharmacists	92 (7.6)
Other or unspecified professionals	246 (20.3)
Students (n=413)	
Medical students	244 (59.1)
Nursing students	69 (16.7)
Pharmacy students	25 (6.1)
Other or unspecified students	75 (18.2)
Age (y; n=1499)	
<25	219 (14.6)
25-34	432 (28.8)
35-44	293 (19.5)
45-54	216 (14.4)
≥55	339 (22.6)

Formal exposure to AI was uncommon. Of the 1366 respondents who answered the item on prior training, only 90 (6.6%) reported having received AI-specific training, whereas 1276 (93.4%) had not. Of the 1175 respondents who answered the training interest item, 920 (78.3%) expressed a desire to receive specific training in AI. Confidence remained limited: of the 1301 respondents who answered the confidence item, only 120 (9.2%) reported being very confident in using AI for diagnostic and patient management support, whereas 407 (31.3%) were not very confident, and 118 (9.1%) were not at all confident (Table 2).

**Table 2.** Training, confidence, and current AI use^a^.

Outcome	Participants, n (%)
Prior AI-specific training (n=1366)	90 (6.6)
Interested in receiving AI training (n=1175)	920 (78.3)
Confidence level in AI use for diagnosis or patient management (n=1301)	
Very confident	120 (9.2)
Somewhat confident	656 (50.4)
Not very confident	407 (31.3)
Not at all confident	118 (9.1)
AI use in professional activity (n=1455)	
Frequent	153 (10.5)
No current use	673 (46.3)

a Denominators vary because the questions were not mandatory; incomplete questionnaires were retained for item-level analyses.

Current use of AI in professional activity was heterogeneous. Of the 1455 respondents who answered the item on use frequency, 673 (46.3%) reported not using AI at all, 297 (20.4%) reported rare use, 311 (21.4%) reported occasional use, 153 (10.5%) reported frequent use, and 21 (1.4%) reported intensive use. Among respondents who reported some AI use and specified contexts of use, the most commonly selected contexts were access to medical information (n=334), help with training (n=277), treatment prescription support (n=203), medical diagnosis (n=201), and medical imaging analysis (n=198).

Perceived benefits of AI included improved diagnosis (774/1625, 47.6%), time savings in care delivery (685/1625, 42.2%), reduction in medical errors (634/1625, 39%), and improved patient follow-up (593/1625, 36.5%). Frequently cited concerns included algorithmic bias (785/1625, 48.3%), transparency and reliability of sources (666/1625, 41%), possible deterioration of the patient–health care professional relationship (628/1625, 38.6%), and data confidentiality (557/1625, 34.3%). Of the 1301 respondents who answered the item on professional threat, 423 (32.5%) considered AI a possible threat to their profession or skills (Table 3).

**Table 3.** Selected reported AI uses, benefits, and concerns (N=1625).

Item	Participants
Contexts of AI use among respondents who reported some AI use and answered this item, n^a^	
Help with access to medical information	334
Help with training	277
Treatment prescription support	203
Medical diagnosis	201
Medical imaging analysis	198
Patient monitoring	117
Expected benefits, n (%)	
Improved diagnosis	774 (47.6)
Time savings in care delivery	685 (42.2)
Reduction in medical errors	634 (39)
Improved patient follow-up	593 (36.5)
Main concerns, n (%)	
Algorithmic bias	785 (48.3)
Limited transparency and reliability of sources	666 (41)
Deterioration of patient–health care professional relationship	628 (38.6)
Data confidentiality	557 (34.3)
Perceived professional threat (n=1301), n (%)	
AI perceived as a threat to their profession or skills	423 (32.5)

a Percentages are not shown for context of use because the item was multi-selection among respondents who reported some AI use.

In exploratory unadjusted analyses, younger age was associated with greater interest in AI training (*P*=.02) and more frequent AI use (*P*<.001). Prior AI training was associated with greater confidence in AI for diagnosis and patient management (*P*=.001). AI use also differed significantly between students and professionals (*P*=.001), and confidence differed across professional groups (*P*=.003), with physicians among the groups reporting the highest confidence levels in the profession-specific comparison (Table 4).

**Table 4.** Exploratory unadjusted associations^a^.

Outcome	Comparison	*P* value	Interpretation
Interest in AI training	Age group	.02	Younger respondents more frequently reported interest in training
Prior AI training	Age group	.31	No significant association
Prior AI training	Student vs professional status	.12	No significant association
Confidence in AI	Prior AI training	.001	Higher confidence among respondents with prior AI training
Current AI use	Age group	<.001	More frequent AI use among younger respondents
Current AI use	Student vs professional status	.001	Distribution of use differed between students and professionals
Confidence in AI	Professional group	.003	Profession-specific confidence differed; physicians were among the most confident groups
Perceived AI as threat	Professional group	.01	Perception of threat differed across professional groups

a All inferential analyses were exploratory, based on Pearson chi-square tests, and unadjusted for potential confounders*.*

## Discussion

### Principal Findings

This national survey found a marked mismatch between the rapid diffusion of AI discourse in health care and the limited formal preparation reported by French health professionals and students. Formal AI-specific training was uncommon, whereas interest in receiving such training was high. Confidence in AI for diagnosis and patient management support remained limited, and current use in professional activity was heterogeneous rather than routine.

When AI was used, it was more often used for supportive or informational purposes than for core diagnostic or therapeutic decision-making. Respondents expected AI to improve diagnosis, save time, reduce errors, and support follow-up, but they also expressed concerns about algorithmic bias, transparency, confidentiality, and the patient–health care professional relationship. These findings suggest that respondents were not opposed to AI in principle but, rather, were conditionally receptive provided that AI remained trustworthy, interpretable, and compatible with safe, human-centered care.

### Comparison With Previous Literature

Earlier studies cited above have already shown that interest in AI frequently exceeds formal preparation across different health care and educational settings [3-6]. The more recent literature further reinforces this pattern and provides additional points of comparison with our French cross-professional sample. Rjoop et al [11] surveyed 394 medical students and pathology trainees in Jordan and found that 66.2% disagreed that their medical schools had educated them about AI, whereas 46.2% expressed interest in learning about AI in medicine; the same study also showed substantial agreement with pathology-specific AI applications, illustrating how perceptions may be shaped by specialty exposure. Syeda et al [12] surveyed 939 undergraduate health care students in Pakistan and reported that only 11.8% had received formal AI training, whereas 78.8% supported AI integration into curricula and 82.2% endorsed AI training as part of medical education. A large international survey of 4596 medical, dental, and veterinary students from 192 faculties in 48 countries similarly found limited AI knowledge, lack of AI courses, and strong desire for more AI teaching [13]. These results are closely aligned with the French pattern observed in our survey, particularly the coexistence of limited formal training and strong educational demand. French data are more limited but point in the same direction. Perrier et al [14] conducted a nationwide survey of 165 young French pediatricians and found favorable attitudes toward AI, but only 5% had received specific AI training, whereas 87% considered AI training necessary. Kotzki et al [15] recently surveyed 388 French health professional students during clinical placements and found that more than half reported using generative AI, mainly for information and documentation support rather than patient-facing activities. Earlier qualitative work by Laï et al [16] based on 40 French stakeholders also suggested that French health professionals were not simply resistant to AI but focused on safe, clinically useful, and responsible implementation. Compared with these French studies, our survey adds a broader cross-professional perspective by including both practicing health professionals and students and assessing use, confidence, perceived benefits, concerns, and training needs within the same national dataset. The broader health service literature also supports the need for this transversal perspective. A 2025 systematic review of 72 empirical studies of health care professionals’ perspectives on AI identified multiple facilitating and hindering factors across individual, interpersonal, institutional, community, and policy levels [17]. The review also noted that many studies focused on radiology or general medicine, limiting the ability to generalize to other fields [17]. Our study therefore complements rather than duplicates the existing literature: it provides a broader national view that is less likely to be driven by the technological culture or implementation history of a single specialty. Specialty-specific European studies further illustrate this issue: the 2024 European Network for the Assessment of Imaging in Medicine and European Society of Medical Imaging Informatics survey found that nearly half of the radiology respondents were already using AI tools, and radiotherapy implementation work has evaluated commercial AI-based auto-contouring systems in clinical practice [18,19].

### Implications for Medical Education and Continuing Professional Development

The clearest educational signal emerging from our survey was the marked gap between the low proportion of respondents who had received formal AI-specific training (90/1366, 6.6%) and the high proportion who wished to receive such training (920/1175, 78.3%). This finding is consistent with the broader international mismatch between the rapid diffusion of AI and insufficient educational preparation. Prior AI-specific training was rare in our study, but interest in training was high. This training gap is highly relevant to medical and health professions education because confidence was greater among those who reported prior AI training. These findings support the integration of structured AI-related competencies across undergraduate, postgraduate, and continuing professional education [7,20,21].

Recent curriculum work suggests that AI training should not be limited to isolated technical exposure. Tolentino et al [7] identified only 2 explicit curriculum frameworks among 21 included papers in their scoping review. Moldt et al [9] further showed that stakeholders expect AI education to include technical knowledge, ethical reasoning, implementation awareness, and clinically meaningful use. Moldt et al [9] recently proposed a postgraduate family medicine AI framework, reinforcing the need for longitudinal, competency-based approaches to AI training [10]. In practical terms, our findings suggest that training should address not only how to use AI tools but also why and when they should be used, how to calibrate trust, and how to recognize situations in which AI may be misleading or clinically unsafe [22,23].

### Ethical, Legal, and Organizational Considerations

The concerns reported by respondents reinforce the importance of framing AI education within broader ethical, legal, and organizational contexts. Concerns about bias, transparency, confidentiality, and the patient–health care professional relationship were prominent in our sample. These concerns are concordant with wider literature showing that bias, explainability, privacy, and implementation barriers are central determinants of trust in health AI [17,24-29].

The organizational implications are also important. Respondents expected AI to support efficiency and safety, yet some also perceived it as a potential threat to professional roles or skills. This tension is understandable because the introduction of new technologies may initially disrupt workflows, increase cognitive load, and create additional work during transition phases, particularly when systems are poorly integrated [26,30,31]. At the same time, AI has been described as having the potential to alleviate part of the administrative and cognitive burden that contributes to professional exhaustion and burnout, thereby helping preserve time for complex decision-making and human interaction [30,32,33]. Our data therefore support a balanced interpretation: clinicians and students expect AI to be helpful but only if it remains interpretable, accountable, and compatible with human-centered care.

### Strengths and Limitations

This study has several strengths. With 1625 respondents, including 1212 (74.6%) practicing health professionals and 413 (25.4%) students, it provides a large French dataset for assessing AI use, concerns, confidence, and training needs across both groups. To our knowledge, it is among the broadest French cross-professional surveys to date on this topic. The questionnaire underwent pilot-testing and exploratory psychometric assessment. A key strength of the present study is its broad and cross-professional sampling frame. Unlike studies focused on a single AI-exposed specialty or one educational subgroup, our survey was designed to capture a wider range of professional and educational backgrounds. This is important because perceptions of AI may be shaped by prior exposure; specialty-specific workflows; and the technological culture of fields such as radiology, radiotherapy, pathology, or other highly digitized disciplines [11,17-19]. By including both practicing health professionals and students, the present study provides a broader national perspective on AI use, concerns, confidence, and training needs in France. This makes the findings particularly relevant for general AI literacy, curriculum design, postgraduate training, and continuing professional development.

Several limitations deserve emphasis. First, this study relied on a convenience sample recruited through a digital professional platform, which may overrepresent respondents already interested in technology. As a result, the findings should not be interpreted as nationally representative. Second, because the exact number of invited platform users was unavailable, a formal response rate could not be calculated. No weighting strategy could be implemented for the same reason. Third, item-level missingness varied across questions; we addressed this by reporting item-specific denominators, but the resulting estimates remain subject to nonresponse bias. Fourth, no formal correction for multiple comparisons was applied because the inferential analyses were exploratory; this increases the risk of type I error and reinforces the need to interpret the reported associations cautiously. Fifth, the psychometric analyses should be interpreted cautiously because exploratory and confirmatory factor analyses were performed on the same overall dataset. Finally, the inferential analyses were unadjusted and exploratory; this study was not designed to support causal interpretation.

### Conclusions

In this national French convenience sample, formal AI training was uncommon despite strong interest in receiving it, and confidence in AI for diagnostic support remained limited. These findings support the relevance of structured AI education across undergraduate, postgraduate, and continuing professional training. They also highlight the importance of integrating ethical, legal, and practical implementation issues into such teaching.

Given the descriptive design and sampling approach, the results should be interpreted as exploratory rather than nationally representative. Future work should include longitudinal and mixed methods studies, particularly studies evaluating how training interventions influence AI literacy, trust calibration, and real-world adoption in clinical environments.

## Supplementary material

10.2196/89152 Multimedia Appendix 1 Artificial Intelligence in Health Care Questionnaire in English and French, with questionnaire development details and exploratory psychometric assessment.
