# Supplementary material for: Use, Concerns, and Perspectives on AI in Health Care Among French Health Professionals and Students: Web-Based Cross-Sectional Survey
Source: JMIR Med Educ. 2026 Aug 5;12:e89152. doi: 10.2196/89152 (PMC13441111; doi:10.2196/89152)
Supplement: Supplementary file 1 [file mededu-v12-e89152-s001.docx]

# Multimedia Appendices

Appendix Section 1. Artificial Intelligence in Health Care Questionnaire (English version)

| **Question** | **Response options** |
| --- | --- |
| You are | Health care professional / Health care student |
| Field of study (if student) | Free text |
| Profession (if professional) | Free text |
| Specialty (if professional) | Free text |
| Practice setting | Hospital / Private clinic / Private practice / Mixed / Other |
| Age group | Under 25 / 25-34 / 35-44 / 45-54 / 55 or more |
| Current use of AI-based solutions in medical or paramedical practice | Intensively / Frequently / Occasionally / Rarely / Not at all |
| If yes, contexts of use (multiple choice) | Medical diagnosis / Medical imaging analysis / Treatment prescription support / Patient monitoring / Administrative-process optimization / Help with training / Help with access to medical information / Epidemic forecasting and public health surveillance |
| Impact of AI on patient management | Rather positive / Rather negative |
| Received specific training on AI | Yes / No |
| Confidence in AI for diagnosis and patient management | Not at all confident / Not very confident / Somewhat confident / Very confident |
| Main concerns related to AI adoption (multiple choice) | Algorithmic bias / Transparency and reliability of sources / Regulations and compliance / Data confidentiality / Degradation of the health professional–patient relationship |
| Could AI represent a threat to your profession or your skills? | Yes / No |
| Main expected benefits of AI in medicine (multiple choice) | Improved diagnosis / Personalized treatments / Time savings in care delivery / Reduction in medical errors / Improved patient follow-up |
| Impact on the caregiver–patient relationship (multiple choice) | More time for the patient / Less time for the patient / Strengthening of trust / Deterioration of trust |
| Tasks that could be assisted or improved by AI (multiple choice) | Diary management / Administrative tasks / Interprofessional communication / Analysis of medical data |
| Would you like specific training in AI? | Yes / No |
| Future of your profession in the face of AI | Potential of AI, need for solid regulations / Concern, but adaptation possible / Optimistic about opportunities / Pessimistic about negative impact / Adaptation necessary, willing to invest in training |

*AI: artificial intelligence.*

Appendix Section 2. Artificial Intelligence in Health Care Questionnaire (French version) Appendix

| **Question** | **Options de réponse** |
| --- | --- |
| Vous êtes | Professionnel de santé / Étudiant en santé |
| Votre filière d’études (si étudiant) | Texte libre |
| Votre profession (si professionnel) | Texte libre |
| Votre spécialité (si professionnel) | Texte libre |
| Votre mode d’exercice | Hôpital / Clinique privée / Cabinet libéral / Mixte / Autre |
| Votre tranche d’âge | Moins de 25 ans / 25-34 ans / 35-44 ans / 45-54 ans / 55 ans ou plus |
| À quelle fréquence utilisez-vous actuellement des solutions d’IA dans votre pratique médicale ou paramédicale ? | Intensivement / Fréquemment / Occasionnellement / Rarement / Pas du tout |
| Si oui, dans quels contextes utilisez-vous l’IA ? (choix multiples) | Diagnostic médical / Analyse d’imagerie médicale / Aide à la prescription / Suivi des patients / Optimisation des processus administratifs / Aide à la formation / Aide à l’accès à l’information médicale / Prévision épidémique et santé publique |
| Comment évaluez-vous l’impact de l’IA sur la prise en charge de vos patients ? | Plutôt positif / Plutôt négatif |
| Avez-vous reçu une formation spécifique sur l’IA ? | Oui / Non |
| Quel est votre niveau de confiance dans l’IA pour l’aide au diagnostic et à la prise en charge ? | Pas du tout confiant / Peu confiant / Plutôt confiant / Très confiant |
| Quelles sont vos principales inquiétudes liées à l’adoption de l’IA en santé ? (choix multiples) | Biais algorithmiques / Transparence et fiabilité des sources / Réglementation et conformité / Confidentialité des données / Dégradation de la relation soignant-patient |
| Pensez-vous que l’IA puisse représenter une menace pour votre profession ou vos compétences ? | Oui / Non |
| Quels sont selon vous les principaux bénéfices attendus de l’IA en médecine ? (choix multiples) | Amélioration du diagnostic / Personnalisation des traitements / Gain de temps / Réduction des erreurs médicales / Amélioration du suivi des patients |
| Quel impact l’IA pourrait-elle avoir sur la relation soignant-patient ? (choix multiples) | Plus de temps pour le patient / Moins de temps pour le patient / Renforcement du lien de confiance / Dégradation du lien de confiance |
| Quelles tâches de votre activité quotidienne pourraient être assistées ou améliorées par l’IA ? (choix multiples) | Gestion de l’agenda / Tâches administratives / Communication interprofessionnelle / Analyse de données médicales |
| Souhaitez-vous recevoir une formation spécifique en intelligence artificielle ? | Oui / Non |
| Comment percevez-vous l’avenir de votre profession face à l’évolution rapide de l’IA en médecine ? | Potentiel de l’IA, besoin d’une réglementation solide / Inquiétude, mais adaptation possible / Optimiste quant aux opportunités / Pessimiste quant à l’impact négatif / Adaptation nécessaire, prêt à investir dans la formation |

**Appendix Section 3. Questionnaire Development and Psychometric Assessment**

The questionnaire was developed through iterative multidisciplinary review by clinicians involved in AI-related clinical care and digital-health implementation. The development process aimed to cover respondent characteristics, current AI use, confidence, perceived benefits and concerns, and interest in training.
Before dissemination, the questionnaire underwent pilot testing with 20 health care professionals and students to assess clarity, wording, and feasibility. This pilot stage was intended as a face-validity and usability check rather than formal standalone validation.
In the analytical sample, internal consistency across domains was acceptable (Cronbach alpha range 0.76-0.84). Sampling adequacy was supported by a Kaiser-Meyer-Olkin index of 0.83, and Bartlett test of sphericity was significant (P<.001). Exploratory factor analysis supported a 3-factor structure (concerns, perceived benefits, and assisted tasks). Confirmatory factor analysis indicated acceptable fit (chi-square/df=2.1, CFI=0.94, TLI=0.92, RMSEA=0.05, SRMR=0.06). Known-groups validity was supported by higher AI confidence among physicians, younger respondents, and those with prior AI training.
More detailed psychometric parameters, including extraction method, rotation, factor-retention criteria, item-retention thresholds, estimator specifications, and omega coefficients, were not retained in a form allowing complete retrospective reporting. Likewise, a formal predefined scoring algorithm for external reuse was not established because the instrument was developed for this exploratory survey.
These psychometric results should therefore be interpreted as exploratory support for the instrument rather than definitive external validation. Pilot testing was limited and exploratory, exploratory and confirmatory analyses were conducted on the same overall dataset, and neither formal content-validity indices nor an external validation sample were available.

Appendix Section 4. Psychometric Summary

| **Construct** | **Number of items** | **Example content** | **Cronbach alpha** | **KMO** | **Bartlett test** | **Factor-loading range** |
| --- | --- | --- | --- | --- | --- | --- |
| Concerns | 5 | Algorithmic bias; transparency; confidentiality | 0.81 | 0.82 | P<.001 | 0.58-0.79 |
| Perceived benefits | 5 | Improved diagnosis; time saving; reduced errors | 0.84 | 0.85 | P<.001 | 0.61-0.83 |
| Assisted tasks | 4 | Administrative support; medical-data analysis | 0.76 | 0.78 | P<.001 | 0.54-0.74 |

*CFI: comparative fit index; KMO: Kaiser-Meyer-Olkin; RMSEA: root mean square error of approximation; SRMR: standardized root mean square residual; TLI: Tucker-Lewis index.*
